# Overexpression of hypoxia-inducible factor-1α and vascular endothelial growth factor in sacral giant cell tumors and the correlation with tumor microvessel density

**DOI:** 10.3892/etm.2014.1971

**Published:** 2014-09-17

**Authors:** SHAOFENG FU, RUI BAI, ZHENQUN ZHAO, ZHIFENG ZHANG, GANG ZHANG, YUXIN WANG, YONG WANG, DIANMING JIANG, DEZHI ZHU

**Affiliations:** 1 Graduate School of Chongqing Medical University, Chongqing 400331, P.R. China; 2 Department of Pediatric Orthopedics, Second Affiliated Hospital of Inner Mongolia Medical University, Huhhot, Inner Mongolian Autonomous Region 010050, P.R. China; 3 Department of Orthopedics, Second Affiliated Hospital of Inner Mongolia Medical University, Huhhot, Inner Mongolian Autonomous Region 010050, P.R. China; 4 Department of Orthopedics, First Affiliated Hospital of Chongqing Medical University, Chongqing 400016, P.R. China; 5 Department of Orthopedics, Fourth Hospital of Inner Mongolia Autonomous Region, Huhhot, Inner Mongolian Autonomous Region 010030, P.R. China

**Keywords:** hypoxia inducible factor-1α, vascular endothelial growth factor, sacral giant cell tumors, microvessel density

## Abstract

Although classified as benign, giant cell tumors of the bone (GCTB) may be aggressive, recur and even metastasize to the lungs. In addition, the pathogenesis and histogenesis remain unclear; thus, the driving factors behind the strong tumor growth capacity of GCTB require investigation. In the present study, the expression levels of hypoxia-inducible factor (HIF)-1α and vascular endothelial growth factor (VEGF), which are promoted by hypoxic conditions, were determined in 22 sacral GCTB samples using immunohistochemistry and western blot analysis. Furthermore, CD34 expression was analyzed using these methods. The correlation between HIF-1α or VEGF expression and the tumor microvessel density (MVD) was then determined. The results demonstrated that HIF-1α, VEGF and CD34 were overexpressed in the 22 sacral GCTB specimens, and overexpression of HIF-1α and VEGF correlated with the tumor MVD. Thus, the present study has provided novel indicators for the tumor growth capacity of GCTBs.

## Introduction

Giant cell tumors of the bone (GCTB) are a rare osteolytic primary bone neoplasm that occur in young adults, characterized by the presence of numerous osteoclasts (1). The majority of GCTBs arise in the metaphyseal-epiphyseal area and are most commonly found in the distal femur, proximal tibia and distal radius (2,3). GCTBs are rarely found in the vertebrae, and the majority of vertebrae GCTBs are located in the sacrum, usually the upper sacrum (4). The sacrum is the fourth most common site, accounting for 1.7–8.2% of cases (5–7). GCTBs also occur in the mobile spine; however, this location only accounts for 2–4% of cases (8,9). In all locations, the neoplasm most commonly occurs between the ages of 20 and 45 years, affecting males and females with equal frequency (9). The pathogenesis and histogenesis remain unclear, since there is no predictable value of histology for the clinical outcome. Although classified as benign, GCTBs are aggressive and recur locally in ≤50% of cases. Up to 5% of GCTBs metastasize to the lungs and spontaneous transformation to a high-grade malignancy occurs in 1–3% of patients (1,10). Recent advances have been made with regard to the pathogenesis of GCTB. The osteoclast differentiation factor, receptor activator of nuclear factor-κB ligand (RANKL), was shown to be highly expressed in stromal cells within GCTBs (11–13), leading to the prediction of the neoplastic ‘driver’ role of stromal cells. In addition, RANKL appears to be critical to the pathogenesis of GCTB. However, more driving factors underlying the strong tumor growth capacity of GCTB require investigation.

Hypoxia has become one of the key issues in the study of tumor physiology. A group of transcription factors have been reported to be involved in the regulation of genes responsible for the metabolic changes under hypoxia (14,15). A pivotal component of these factors is hypoxia-inducible factor (HIF)-1, a heterodimer consisting of an oxygen-sensitive HIF-1α subunit and a constitutively expressed HIF-1β subunit (16). HIF-1 binds to a conserved DNA consensus on the promoter region of target genes, known as hypoxia-responsive elements (17–19). HIF induces a vast array of gene products, which control cellular processes that are crucial for hypoxic adaptation (20). HIF-1 is a key regulator of vascular endothelial growth factor (VEGF) and other angiogenic factors (21,22), which play crucial roles in the growth and progression of solid tumors (23–26).

When GCTBs are involved with the sacrum, patients present with localized lower-back pain that may radiate to one or both lower limbs. Neurological symptoms, if present, are often subtle (27). Vague abdominal discomfort, early satiety and a change in bowel/bladder habits are possible. Due to the generally insidious onset of symptoms in patients with sacral GCTBs, the tumor usually grows to a large size prior to diagnosis; thus, may undergo hypoxia. However, little is known with regard to the expression of HIF-1α and VEGF in GCTBs, particularly in sacral GCTBs.

In the present study, the expression levels of HIF-1α and VEGF were quantitatively determined in 22 sacral GCTB samples using immunohistochemical methods. In addition, to provide novel indicators for the degree of malignancy and the prognosis of GCTBs, correlations between HIF-1α or VEGF expression with the invasion and recurrence were assessed.

## Materials and methods

### Tissue samples and ethical approval

Use of the 22 sacral GCTB samples was approved by the Internal Review Board of the Department of Orthopedics, First Affiliated Hospital of Chongqing Medical University (Chongqing, China). The samples were surgical resections from patients with sacral GCTBs registered in the aforementioned hospital between January 1998 and December 2012. A total of 10 normal sacral samples were used as a control. All the tissue samples used for immunohistochemical staining were formalin-fixed and paraffin-embedded following surgical resection, while the tissue samples used for immunoblotting were frozen at −80°C immediately after surgical resection. Hematoxylin-eosin slides, pathology reports, other medical records and treatment procedures were reviewed and standardized to ensure study homogeneity. The specimens used in the study were human sacral GCTB specimens removed by surgery as part of the cancer treatment. Prior to surgery, the patients granted consent for the use of the excised cancer tissue in medical or scientific research.

### Immunohistochemical staining

GCTB sample slides were deparaffinized by heating at 55°C for 30 min, washed with xylene and rehydrated serially in 100, 90 and 70% ethanol and phosphate-buffered saline (PBS). Antigen retrieval was performed by heating for 20 min at a constant temperature of 98°C in 10 mM sodium citrate (pH 6.0; 250 ml), and endogenous peroxidase activity was inhibited with 0.3% hydrogen peroxide for 20 min. Rabbit polyclonal antibodies against HIF-1α and VEGF (Abcam, Cambridge, UK), and a mouse monoclonal antibody against CD34 (Abcam) were used to perform the immunohistochemical assay. The antibodies were diluted 1:50 with goat serum separately. Following incubation with the primary antibodies at room temperature for 1 h, the sections were washed with PBS three times for 5 min each, and incubated with a goat anti-rabbit/mouse immunoglobulin G horseradish peroxidase (HRP)-conjugated secondary antibody (Abcam). Following an additional three washes, 3,3′-diaminobenzidine HRP substrate (Abcam) was added for 1 min and counterstained with Mayer’s hematoxylin. The samples were then dehydrated and sealed with cover slips. Negative controls were performed by omitting the primary antibodies. A semi-quantitative system was used to analyze the level of antigen expression: Immunoreactivity was scored as either negative (0), focal (1+; <25% positive cells), moderate (2+; 25–50% positive cells) or diffuse (3+; >50% positive cells). The intensity of immunostaining was rated as follows: None (0), weak (+1), moderate (+2) and intense (+3). The immunohistochemistry score was defined as the sum of the aforementioned two scores. Specimens were analyzed by two observers and scored following a consensus by the observers (28).

### Semi-quantitative immunoblotting

Tissue samples for immunoblotting were placed in 10 ml ice-cold isolation solution, containing 250 mM sucrose, 10 mM triethanolamine (Sigma-Aldrich, St. Louis, MO, USA), 1 mg/ml leupeptin (Sigma-Aldrich) and 0.1 mg/ml phenylmethylsulfonyl fluoride (Sigma-Aldrich) titrated to pH 7.6, and the mixture was homogenized at 13,600 × g with three strokes for 15 sec using a tissue homogenizer (PowerGun 125; Thermo Fisher Scientific, Pittsburgh, PA, USA). Following homogenization, the total protein concentration was measured using a bicinchoninic acid protein assay reagent kit (Thermo Fisher Scientific, Rockford, IL, USA), which was adjusted to 2 mg/ml with isolation solution. Equal amounts of protein and sample buffer were separated using 12% gradient SDS-PAGE, stained with Coomassie Brilliant Blue and transferred to a polyvinylidene fluoride membrane. The blotted membrane was blocked with Tris-buffered saline containing 5% milk, and incubated with HIF-1α, VEGF or CD34 rabbit polyclonal antibodies (1:500; Santa Cruz Biotechnology, Inc., Santa Cruz, CA, USA), followed by incubation with a HRP-coupled secondary antibody (1:1000; Cell Signaling Technology, Inc., Danvers, MA, USA). The proteins were detected using enhanced chemiluminescence (Thermo Fisher Scientific). All immunoblots were representative of at least three independent experiments.

### Calculation of the tumor microvessel density (MVD)

At a low-power field (magnification, ×200), the tumor tissue sections were screened and five areas with the most intense neovascularization (hot spots) were selected. Microvessel counts of these areas were performed at a high-power field (magnification, ×400). Any CD34 positive endothelial cell or endothelial cell cluster clearly separated from adjacent microvessels, tumor cells and connective tissue elements were considered to be single countable microvessels. Branching structures were counted as one, unless there was a break in the continuity of the vessel, in which case the structure was counted as two distinct vessels. Three fields per tumor section were counted in the areas that appeared to contain the greatest number of microvessels on scanning at low magnification. MVD was defined as the mean score from the five fields.

### Statistical analysis

Statistical analyses were performed using SPSS 16.0 software (SPSS, Inc., Chicago, IL, USA). HIF-1α, VEGF and CD34 expression levels between the two groups were analyzed using the Student’s t-test. Correlations between HIF-1α or VEGF expression and the MVD value were analyzed using Spearman’s rank correlation. P<0.05 was considered to indicate a statistically significant difference.

## Results

### High expression levels of HIF-1α and VEGF in the sacral GCTB samples

**Figure 1:**
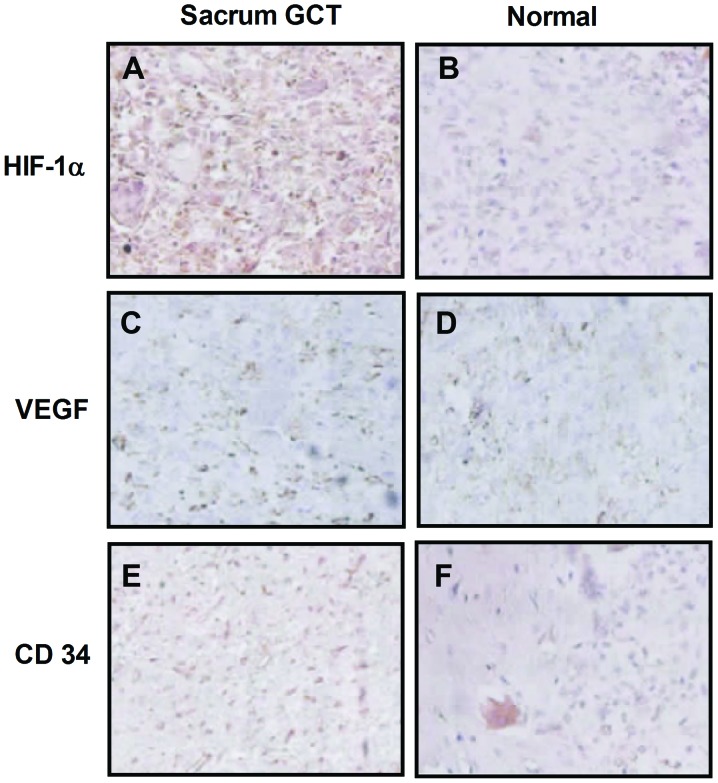
Representative immunohistochemical staining for HIF-1α, VEGF and CD34 expression in (A, C and E) sacral GCT samples, respectively, and (B, D and F) normal sacral tissues, respectively (magnification, ×200; light microscopy). HIF-1α, hypoxia-inducible factor 1α; VEGF, vascular endothelial growth factor; GCT, giant cell tumor.

HIF-1α and VEGF expression levels were determined using immunohistochemical staining in the sacral GCTB samples. The results demonstrated that HIF-1α was primarily located in the cytoplasm of the mononuclear stromal cells and rarely located in the tumor cell nuclei, as shown in Fig. 1A and B. VEGF was also located in the cytoplasm of mononuclear stromal cells or multinucleated giant cells. Immunohistochemical staining revealed that more HIF-1α-positive cells were observed in sacral GCTB samples compared with normal sacral tissues (Fig. 1C and D). In addition, more mononuclear stromal cells and multinucleated giant cells were VEGF-positive in sacral GCTB specimens compared with normal tissues.

**Figure 2:**
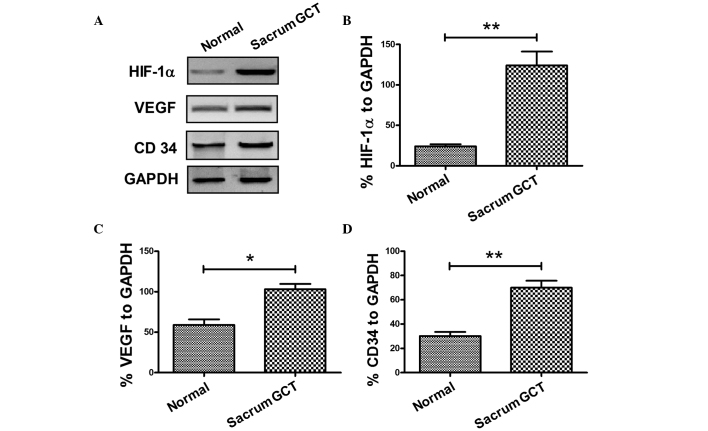
Representative western blot analysis for HIF-1α, VEGF and CD34 expression in sacral GCT specimens. (A) HIF-1α, VEGF and CD34 protein expression levels were determined in sacral GCT and normal samples (n=22) using western blot analysis. Relative expression levels of (B) HIF-1α, (C) VEGF and (D) CD34 against GAPDH in the sacral GCT samples. ^*^P<0.05 and ^**^P<0.01. HIF-1α, hypoxia-inducible factor 1α; VEGF, vascular endothelial growth factor; GCT, giant cell tumor.

To confirm the expression of HIF-1α and VEGF in sacral GCTB samples, the protein expression levels were analyzed using western blot analysis. As shown in Fig. 2, HIF-1α and VEGF were overexpressed in sacral GCTB specimens when compared with the normal sacral tissues. The mean relative expression of HIF-1α against GAPDH in the sacral GCTB specimens was 124.00±17.20%, while the mean value in the normal sacral tissues was 24.20±2.60% (Fig. 2A and B), which produced a 95% confidence interval (CI) of 59.68–139.9 and a statistically significant difference (P<0.01). The mean relative expression of VEGF in the sacral GCTB specimens was 103.00±6.63%, while the mean value in the normal sacral tissues was 59.00±6.78% (Fig. 2A and C), which had a 95% CI of 22.12–65.88 and a statistically significant difference (P<0.05). Therefore, HIF-1α and VEGF were overexpressed in the sacral GCTB samples as compared with the normal sacral tissues.

### Determination of the tumor MVD value in the sacral GCTB samples

HIF-1 and VEGF are known to have key regulatory roles in the vascular endothelial growth and angiogenesis of tumors (21–26). To determine the effect of HIF-1α and VEGF on sacral GCTB angiogenesis, the MVD of sacral GCTB samples was determined using immunohistochemical staining and western blot analysis, from which the correlations between HIF-1α or VEGF expression with the intratumoral MVD value were analyzed. Firstly, the expression of CD34, a molecular marker of vascular endothelial cells, was determined and the MVD value in the sacral GCTB samples was calculated. As shown in Fig. 1E, CD34 expression was primarily located in the vascular endothelial cells surrounding the sacral GCTB. Western blot analysis was also performed to determine CD34 expression in the sacral GCTB samples. The mean relative expression of CD34 in the sacral GCTB specimens was 70.00±5.70%, while the mean value in the normal sacral tissues was 30.20±3.26% (Fig. 2A and D), which produced a 95% CI of 24.65–54.95 and a statistically significant difference (P<0.01). Thus, overexpression of CD34 was confirmed in the sacral GCTB samples.

### Overexpression of HIF-1α or VEGF is correlated with a high MVD value in the sacral GCTB samples

**Figure 3:**
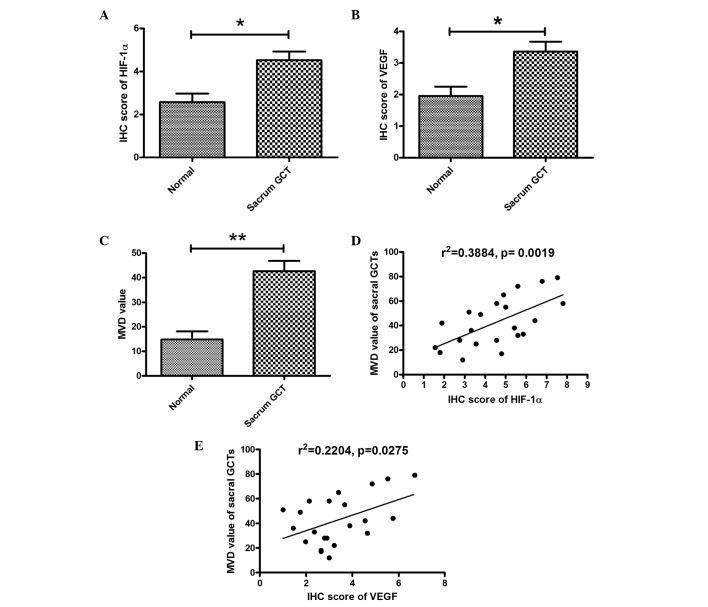
Overexpression of HIF-1α and VEGF correlates with an increased MVD value in sacral GCT specimens. Immunohistochemistry scores of (A) HIF-1α and (B) VEGF overexpression in the sacral GCT specimens. (C) Increased MVD values were observed in sacral GCT specimens. Correlations between the relative (D) HIF-1α and (E) VEGF expression levels with the MVD value. P<0.05 was considered to indicate a statistically significant difference. MVD, microvessel density; GCT, giant cell tumor; HIF-1α, hypoxia-inducible factor 1α; VEGF, vascular endothelial growth factor.

To investigate the correlation between HIF-1α and VEGF expression levels and the MVD of the sacral GCTB samples, the immunohistochemical staining results of HIF-1α and VEGF expression were semi-quantitatively interpreted by calculating the immunohistochemistry score of each molecule in each sacral GCTB sample. The mean immunohistochemistry score of HIF-1α in the sacral GCTB samples was 4.53±0.40 compared with 2.58±0.39 in the control samples (P=0.03); the mean immunohistochemistry score of VEGF in the sacral GCTB specimens was 3.36±0.31 compared with 1.95±0.30 in the control samples (P=0.049). The correlation between HIF-1α and VEGF expression in the sacral GCTB samples with the MVD value was then determined. As shown in Fig. 3, a positive correlation was observed between the HIF-1α or VEGF expression and the MVD value in the GCTB samples (Fig. 3D and E). Therefore, overexpression of HIF-1α or VEGF was confirmed to be correlated with a high MVD value in the sacral GCTB samples.

## Discussion

GCTB is a benign neoplasm characterized by the presence of mononuclear cells, together with multinucleated giant cells that resemble normal osteoclasts (29). GCTB may exhibit considerable local aggressiveness, often associated with intense osteolytic activity. In a small number of cases, GCTBs may develop lung metastases, indicating that specific tumors may acquire an aggressive phenotype (30). HIF mediates the pathophysiological response to hypoxia in ischemic diseases, including various types of cancer (31). Knowles *et al* (32) first described HIF expression in GCTB and human osteoclasts in culture and *in vivo*. The authors proposed a model whereby HIF-dependent VEGF secretion from stromal cells mediates paracrine effects to stimulate osteoclast differentiation (32).

In the present study, a total of 22 sacral GCTB samples were collected, and HIF-1α and VEGF expression levels were determined using immunohistochemical staining and western blot analysis. Significantly high levels of HIF-1α and VEGF expression were confirmed in the GCTB samples using the two methods. In addition, the expression of CD34, an MVD marker, was determined using immunohistochemical staining and western blot analysis. CD34 was also found to be significantly overexpressed in the sacral GCTB samples. Furthermore, Spearman’s rank correlation analysis demonstrated significant correlations between HIF-1α or VEGF expression and the MVD value in the GCTB samples.

HIF-mediated induction of VEGF is known to have a number of effects, including the recruitment of monocytes and osteoclasts in GCTBs (33, 34) and supporting osteoclast survival and activity (35). Local hypoxia has been shown to correlate with HIF-1α expression in osteoblasts, local VEGF production and increased numbers of tartrate-resistant acid phosphatase-positive osteoclasts (36). However, hypoxia and growth factors function indirectly on osteoclasts via the promotion of paracrine secretion of osteoblast-derived VEGF. Osteoclasts in culture and osteoclast-like giant cells *in vivo* were shown to express HIF-1α and HIF-2α, which further induced the expression of VEGF and other downstream genes.

The results of the present study indicated that hypoxia and subsequent induced growth factors within the bone microenvironment may contribute to the initiation and development of GCTB. Local hypoxia may promote the production of HIF and VEGF (37,38) and (pre)-osteoclast recruitment. Within established tumors, hypoxia comprises chronic diffusion-limited hypoxia due to inadequate tumor vasculature (39), acute hypoxia due to perfusion fluctuation (40) and metabolic hypoxia due to fluctuations in the rate of oxygen utilization (41). Despite GCTB being highly vascular, it is likely that HIF expression within these tumors is driven by hypoxia, as well as microenvironmental growth factors.

In conclusion, the present study demonstrated that HIF-1α, VEGF and CD34 are overexpressed in sacral GCTBs using immunohistochemistry and western blot analysis. The MVD value, calculated using CD34 expression, was also shown to be upregulated in sacral GCTBs, and significantly correlate with HIF-1α or VEGF expression in these GCTB samples. Therefore, the present study has provided novel indicators for the tumor growth capacity of GCTBs.
